# Cross-talk between exercises and renin-angiotensin-aldosterone-system blockade in hypertension

**DOI:** 10.1038/s41440-024-01688-6

**Published:** 2024-05-15

**Authors:** Elena Kaschina

**Affiliations:** Charité - Universitätsmedizin Berlin, Institute of Pharmacology, Max Rubner Center for Cardiovascular Metabolic Renal Research (MRC), Berlin, Germany

**Keywords:** RAAS, Losartan, Exercise, Hypertension, SHR

Antihypertensive medications remain the most effective treatment for blood pressure (BP) reduction. The drugs affecting the renin-angiotensin-aldosterone-system (RAAS), the ACE inhibitors and the AT1 receptor antagonists, are highly recommended as first line therapies [1]. Recent Hypertension Guidelines also emphasize the role of lifestyle modifications which can enhance the effects of antihypertensive treatment [1]. In particular, regular aerobic and resistance exercise are recommended for prevention and treatment of hypertension for patients with high-normal BP and hypertension without additional major risk factors.

In this view, a combination of pharmacological and exercise interventions may be beneficial due to their synergistical or additive mechanisms of action. Unfortunately, studies on the effectiveness of such combined therapies are rare.

In this issue Aguilar and colleagues [2] have shown in an elegant experimental study the benefits of combined treatment with the AT1 antagonist losartan and aerobic physical training. The experiments were performed in spontaneously hypertensive rats (SHR), which are known to develop pre-hypertension and hypertension mainly due to increased vascular resistance and autonomic dysfunction [3]. In this study, 18-week-old rats with established hypertension were trained through swimming for a period of 12 weeks. The exercise group was compared with losartan treated rats and with animals that were treated with combination of losartan and exercise. In line with previous data by Vieira et al. [4], physical training alone reduced BP in SHRs. However, Schlüter et al. [3], based on a meta-analysis of 18 studies, had previously concluded that the exercise per se did not reduce BP in SHR with established hypertension. According to this meta-analysis, BP pressure reduction was observed only in rats that started exercises in the pre-hypertensive or very early hypertensive state. Indeed, the controversy between the studies could be explained by different training protocols and methods of BP measurement. Thus, further research is needed.

In the present study Aguila and colleagues [2] have also shown that exercise training potentiates the BP lowering effect of the AT1 receptor antagonist losartan in SHRs. In contrast, in young 8-week-old SHR rats, Azevedo et al. could not find a synergistic effect of exercise training via treadmill on BP when combined with losartan [5]. This points again to the role of disease stage and exercise training protocol for the outcome of the study.

In the present study, the authors further characterized the pathophysiological mechanisms underlying BP regulation under losartan and exercise regime [2]. They observed, in concurs with previous data (reviewed in Masson GS and Michelini LC [6]), an improvement of autonomic balance after training, namely a reduction of sympathetic and an increase of vagal tone. Additional sympathetic tone reduction by losartan contributed to improved autonomic balance and by this mechanism to BP lowering as well. Losartan reduced cardiac hypertrophy, confirming the well-known effect of the AT1 receptor antagonists [7, 8], whereas exercise, on the contrary, increased left ventricular thickness. As a result, a combination of losartan with exercises ameliorated left ventricular remodeling. This finding is in line with the study by Tomaz de Castro et al. [9], who showed that losartan treatment together with exercise reduced cardiomyocyte diameter and decreased MMP2 activity in the heart coinciding with improved cardiac parameters. Thus, besides BP lowering, combined treatment may delay the development of adverse cardiac remodeling.

Aguila and colleagues also claimed that exercise training - but not losartan - improved cardiac contractility in SHRs [2]. This conclusion goes against of results from previous study by Gohlke et al. [7], who demonstrated an increased cardiac contractility on losartan treatment in SHRs. Indeed, as discussed by the authors, the controversy between these studies could be explained by different age of rats and, consequently, the stage of hypertension as well as by different dose of losartan.

Altogether, Aguilar and colleagues have shown that the BP benefits, which result from exercise training in rats with established hypertension, are partly comparable with antihypertensive therapy and a combination of exercises with AT1 receptor blockade is more effective than the interventions alone. Despite some limitations, the findings of Aguilar and colleagues are novel and exciting and indeed contribute to our knowledge on possible cross-talk between RAAS and exercises in hypertension. These interactions are complex and occur on neurohumoral, hemodynamic and metabolic levels (Fig. 1).

**Fig. 1:**
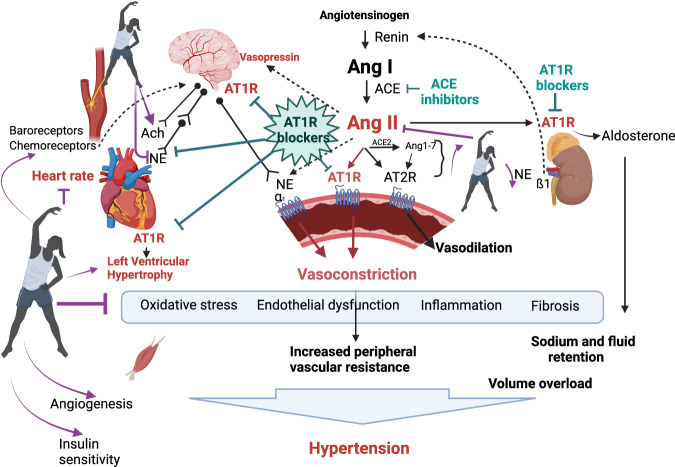
Cross-talk between Exercises and Renin-Angiotensin-Aldosterone-System Blockade in Hypertension. Ang I angiotensin I, Ang II angiotensin II, Ang 1-7 angiotensin 1-7, AT1R angiotensin type 1 receptor, AT2R angiotensin type 2 receptor, ACE angiotensin converting enzyme, NE norepinephrine, Ach acetylcholine, α_1_ alpha1 adrenoreceptor, ß_1_ beta1 adrenoreceptor

RAAS contributes to pathological changes in the cardiovascular and central nervous system, leads to vasoconstriction, sodium and water retention, oxidative stress, inflammation, fibrosis, and hypertrophy and thus to development of pre-hypertension and hypertension. Pharmacological blockade of this system prevents the development of hypertension and protects end organs [8].

The benefits that exercise exerts on BP lowering are explained by the interactions with neurohumoral systems, in particular with the sympathetic nervous system and the RAAS. Importantly, exercises may directly interfere with the RAAS, and thus, potentiate the effects of antihypertensive RAAS blockers. For example, aerobic exercises are known to reduce RAAS hyperactivity by decreasing Angiotensin II levels and in turn the AT1 receptor activation. In parallel, physical training may stimulate protective ACE2-Ang-(1-7)-Mas receptor axis pathway, followed by additional vasodilation through AT2 receptor activation and decreased peripheral vascular resistance (reviewed in Teles et al. [10]).

Furthermore, moderate exercise training in hypertensive rats improves cardiovascular autonomic balance and reduces cardiac sympathetic tone after training, thus contributing to vasodilatation, reduction in peripheral vascular resistance and decreased heart rate [6]. The reduction of sympathetic drive and an increase of vagal tone after training has been evidenced in the present study by Aguilar and colleagues [2]. Reduced sympathetic tone, that is followed by down-regulation of renin secretion, is also known to decrease the synthesis of Angiotensin II.

In addition, lower peripheral vascular resistance in SHRs after exercise might be related to decreased oxidative stress, improved endothelial function via enhanced NO synthesis and reduced inflammation in the vessels as well as beneficial elastin and collagen content [10].

Moreover, exercise as well as an inhibition of RAAS, contributes to brain, heart, kidney and muscle protection in hypertensive rats by inhibiting the inflammatory and fibrotic pathways and by reducing oxidative stress and apoptosis [8, 10]. Again, both exercise training and ACE inhibition activate angiogenesis in SHRs, and when applied together these interventions enhance myocardial capillarization even more [11].

In view of discussed cross-talk mechanisms between exercises and the RAAS, physical training seems to be a potential non-pharmacological adjuvant therapy to antihypertensive medication, in particular to RAAS blockade.

Further studies need to be conducted to investigate the effects of exercise training and its combination with other classes of antihypertensive drugs, in various animal models of hypertension, in different stages of disease and variations in exercise intensity, as well as in different genders.

## References

[1] Unger T, Borghi C, Charchar F, Khan NA, Poulter NR, Prabhakaran D (2020). 2020 International society of hypertension global hypertension practice guidelines. Hypertension.

[2] Aguilar BA, Vieira S, Oliveira ACV, da Silva JVMB, Paixao TV, Rodrigues KP, et al. Physical exercise is essential for increasing ventricular contractility in hypertensive rats treated with losartan. Hypertension Res. 2024. 10.1038/s41440-024-01611-z. 10.1038/s41440-024-01611-z 38418900

[3] Schlüter KD, Schreckenberg R, da Costa Rebelo RM (2010). Interaction between exercise and hypertension in spontaneously hypertensive rats: a meta-analysis of experimental studies. Hypertens Res.

[4] Vieira S, Aguilar BA, Veiga AC, Philbois SV, Freitas ACS, Rodrigues KP (2022). Integrative physiological study of adaptations induced by aerobic physical training in hypertensive hearts. Front Physiol.

[5] Azevedo LF, Brum PC, Mattos KC, Junqueira CM, Rondon MU, Barretto AC (2003). Effects of losartan combined with exercise training in spontaneously hypertensive rats. Braz J Med Biol Res.

[6] Masson GS, Michelini LC (2017). Experimental evidences supporting training-induced benefits in spontaneously hypertensive rats. Adv Exp Med Biol.

[7] Gohlke P, Linz W, Schölkens BA, Wiemer G, Unger T (1996). Cardiac and vascular effects of long-term losartan treatment in stroke-prone spontaneously hypertensive rats. Hypertension.

[8] Kaschina E, Unger T. Prehypertension and the renin angiotensin aldosterone system. in: hypertension and cardiovascular protection, prehypertension and cardiometabolic syndrome (Reuven Zimlichman et al. Eds.) Springer, 2017, 978-3-319-75309-6, 439324_1_En, (22).

[9] Tomaz de Castro QJ, Araujo CM, Watai PY, de Castro E Silva SS, de Lima WG, Becker LK (2021). Effects of physical exercise combined with captopril or losartan on left ventricular hypertrophy of hypertensive rats. Clin Exp Hypertens.

[10] Teles MC, Portes AMO, Coelho BIC, Resende LT, Isoldi MC (2023). Cardiac changes in spontaneously hypertensive rats: modulation by aerobic exercise. Prog Biophys Mol Biol.

[11] Zioada AM, Hassan MO, Tahlikar KI, Inuwa IM (2005). Long-term exercise training and angiotensin-converting enzyme inhibition differentially enhance myocardial capillarization in the spontaneously hypertensive rat. J Hypertens.

